# Computational docking and *in vitro* analysis identifies novel arylidene analogue FPMXY-14 against renal cancer cells by attenuating Akt

**DOI:** 10.32604/or.2022.03570

**Published:** 2022-08-01

**Authors:** HASSAN M. OTIFI, MISHARI ALSHYARBA, MAJED AL FAYI, AYED A. DERA, PRASANNA RAJAGOPALAN

**Affiliations:** 1 Department of Pathology, College of Medicine, King Khalid University, Abha, Saudi Arabia; 2 Department of Surgery, College of Medicine, King Khalid University, Abha, Saudi Arabia; 3 Department of Clinical Laboratory Sciences, College of Applied Medical Sciences, King Khalid University, Abha, Saudi Arabia; 4 Central Research Laboratory, College of Applied Medical Sciences, King Khalid University, Abha, Saudi Arabia

**Keywords:** Arylidene indanone, Apoptosis, Renal cancer, Akt, Flow cytometry

## Abstract

Targeted therapies are gaining global attention to tackle Renal Cancer (RC). This study aims to screen FPMXY-14 (novel arylidene analogue) for Akt inhibition by computational and *in vitro* methods. FPMXY-14 was subjected to proton NMR analysis and Mass spectrum analysis. Vero, HEK-293, Caki-1, and A498 cell lines were used. Akt enzyme inhibition was studied with the fluorescent-based kit assay. Modeller 9.19, Schrodinger 2018-1, LigPrep module, and Glide docking were used in computational analysis. The nuclear status was assessed by PI/Hoechst-333258 staining, cell cycle, and apoptosis assays were performed using flow cytometry. Scratch wound and migrations assays were performed. Western blotting was applied to study key signalling proteins. FPMXY-14 selectively inhibited kidney cancer cell proliferation with GI_50_ values of 77.5 nM and 101.40 nM in Caki-1 cells and A-498 cells, respectively. The compound dose-dependently inhibited Akt enzyme with an IC_50_ value of 148.5 nM and bound efficiently at the allosteric pocking of the Akt when computationally analyzed. FPMXY-14 caused nuclear condensation/fragmentation, increased the sub G_0_/G_1_, G_2_M populations, and induced early, late phase apoptosis in both cells when compared to controls. Treatment of the compound inhibited wound healing and migration of tumor cells, while proteins like Bcl-2, Bax, and caspase 3 were also altered. FPMXY-14 effectively inhibited the phosphorylation of Akt in these cancer cells, while total Akt was unaltered. FPMXY-14 exhibited anti-proliferative and anti-metastatic activities in kidney cancer cells by attenuating the Akt enzyme. Further pre-clinical research on animals with a detailed pathway elucidation is recommended.

## Introduction

Renal Cancer (RC) constitutes the 9^th^ most common type of cancer in the male population [1] and stands fourteenth among all cancer incidences around the world [2]. For the year 2018, a global statistical analysis reports over four million new cases of RC, while mortality was reported to be over 1.7 million [1]. Another statistical report records 1.76 million new RC cases, with 0.6 million deaths in the USA for 2019 [3]. In the Kingdom of Saudi Arabia, the average age of the patients diagnosed with RC types was around fifty-six, where 61% of the RC cases were males [2]. It was indicated that the incidence of kidney cancer cases in the Kingdom was increasing at an alarming rate and associated with other risk factors [4]. Of many forms, Renal Cell Carcinoma (RCC) is the most frequent type, accounting for 95% of all kidney cancer types [5]. Inadequate early symptoms, chemotherapeutic resistance, and high complexity of clinical manifestations are the main challenges in the treatment regimen of RCC, which leads to a very poor prognosis of less than 5% of the overall five-year survival rate [3].

Targeted therapies have been gaining global attention recently due to their pragmatic success from the bench into clinical practice. The success of these therapies preliminary lies on their specific targets of cancer cells while sparing the non-cancerous normal cells. Protein kinases are one among such crucial targets since they play a vital role in driving numerous cellular functions via substrate polymerizations [4]. Akt, otherwise known as Protein kinase-B, is one such serine-threonine kinase that plays a crucial role in cell growth, cell division, and apoptosis [5]. Abnormalities in the expression of Akt are widely reported in several human cancers [6], which has made Akt one of the prime choices as a drug target in controlling various cancer types.

Recent developments in medicinal chemistry have contributed to an array of small synthetic molecules directed to be tested for targeted efficacy against many forms of cancers [7,8]. Emerging hits obtained from basic screening protocols within these small molecules add to the potential value in current drug research to focus on less toxicity and economical drugs. Indane-1-ones are a class of small molecules with high drug-likeliness properties [9]. Several studies have proven Arylidene indan-1-ones to be effective against primary and resistant forms of cancers [10–12]. Therefore, the current investigation aims to screen one novel arylidene indanone analogue (FPMX-14) as an Akt inhibition target against renal carcinoma cells using computation and *in vitro* models.

## Materials and Methods

### Materials

Unless specified, all chemicals and reagents used in this study were procured from Sigma (St. Louis, MO, USA). A498, Caki-1, HEK-293, and Vero cell lines were obtained from the American Type Culture Collection (ATCC, MA, USA). Z’-LYTE Kinase Assay Kit was from Thermo Fischer Scientific (MA, USA). Cell cycle assay reagent, Transendothelial migration assay kit, and Annexin V kit were obtained from Millipore Corp (MA, USA). Unless specified, all antibodies for western blotting were purchased from Abcam, Corp, UK. Bax, Bcl-2, Caspase-3, p-Akt (ser 473), t-Akt, and beta-actin antibodies were purchased from Santacruz biotechnology, Santacruz (CA, USA).

### Methods

#### Chemical synthesis and characterization of FPMXY-14

Synthesis of 2-(4-isopropyl-benzylidene)-4,7-dimethyl-indan-1-one was achieved in two steps. In the first step, Baylis–Hillman adduct of 4-isopropyl-benzaldehyde and t-butyl acrylate treated with DABCO in presence of silica gel [>200 mesh)] were added. The reaction mixture was kept at room temperature. The reaction was monitored by thin-layer chromatography. After an appropriate time, ethyl acetate was added, stirred, and filtered. The solid silica gel was washed with ethyl acetate. The combined organic layer was dried over anhydrous sodium sulphate, and the solvent was removed by distillation. The crude compound was purified by column chromatography (silica gel, 5% ethyl acetate in hexane) to give 2-(Hydroxy-(4-isopropyl-phenyl)-methyl) acrylic acid tert-butyl ester.

To the above hydroxy ester and p-xylene, a catalytic amount of Conc. H_2_SO_4_ was added and refluxed for 3 h. The solvent p-xylene was removed under reduced pressure, and the residue was treated with trifluoroacetic anhydride in 1,2-dichloroethane and refluxed for 2 h. The reaction mixture was diluted with ether and washed with an aqueous solution of NaHCO_3_. The organic layer was dried over anhydrous sodium sulphate, and the solvent was evaporated. The product was purified by using column chromatography (silica gel, 5% ethyl acetate in hexane) followed by crystallization (chloroform and hexane, 2:3). to give 2-(4-isopropyl-benzylidene)-4,7-dimethyl-indan-1-one. The synthesized compound was subjected to proton NMR analysis and Mass spectrum analysis to determine molecular formula, structure, IUPAC name, and molecular weight.

#### Cell culture

Vero, HEK-293, and A498 cells were cultured in Eagle’s Minimum Essential Medium (ATCC). Caki-1 cells were cultures in ATCC-formulated McCoy’s 5a Medium (ATCC). HUVEC cells were maintained in F-­12K full growth Medium containing 0.1 mg/ml heparin and 0.05 mg/ml endothelial cell growth supplement (ECGS); at a final concentration of 10% fetal bovine serum (FBS). The full growth medium was supplemented with 10% FBS, 100 U/ml of penicillin, and 100 U/ml streptomycin. Cells were maintained in a humidified atmosphere of 5% CO_2_ incubator at 37°C, and assays were performed when the cells were approximately 70% confluent.

#### Cell proliferation assay

Cell proliferation was analyzed and performed as described elsewhere [13] with some modification. Normal or cancer cells were seeded in a 96-well plate at 5000 cells/well in a respective growth medium. After adhering to the base, different concentrations of FPMXY-14 or sunitinib were added and incubated at 37°C and 5% CO_2_. At the end of 72 h incubation, 5 mg/ml MTT reagent in 25 µl was added and incubated for 4 h. The media aspired, and the colour formazan product was dissolved in Dimethyl sulphoxide (DMSO). Contents were transferred to 96 well-clear bottom plates, and absorbance was recorded at 560 nM with 640 nM as a reference wavelength. The percentage of cell proliferation inhibition were calculated by subtracting day 0 values, and results were analyzed using Graph pad Prism 6.0 software (La Jolla, CA, USA).

#### Akt enzyme assay

Akt enzyme assay was performed with Z’-LYTE™ kit, a fluorescence-based, coupled-enzyme format as per the manufacturer’s instructions. Staurosporine was used as a positive control. Results of the mean values were plotted in GraphPad prism software, 6.0 (La Jolla, CA, USA), and linear regression analysis was used to determine the IC_50_ values.

#### Molecular docking

Akt structure was used as per the previously described method [14]. In brief, crystal structure (PDB # 4ejn) was obtained, and missing loop and side chains were built using Modeller 9.19 and further refined using the protein preparation wizard module of Schrodinger 2018-1. Prior to optimizing the structure with the OPLS-3 forcefield, the bond orders were allocated, and the missing hydrogens were added and applied for docking studies. Ligands were prepared using the LigPrep module from the Schrodinger suite. Glide docking was performed using standard precision mode from the Glide module [15] of the Schrodinger suite. The grid box was focused on the allosteric pocket of the Akt enzyme as described elsewhere [16]. LigPrep module was used to prepare the ligands and docked in the generated grid.

#### MM/GBSA

The glide docking pose was further used to quantify the binding energy interaction. The binding energies were calculated using the prime MM-GBSA module from Schrodinger Suite. The scores obtained from MM-GBSA were considered using default parameters via the VSGB solvation model [17].

#### Dual nuclear staining

Analysis of the nuclear status after FPMXY-14 was carried out using Propidium iodide/Hoechst 333258 dual staining as described elsewhere [18] with some modifications. Caki-1 or A-498 cells at 1 × 10^5^ cells were seeded in 6 well plates containing sterile coverslips and allowed to adhere for 24 h. 75 nM and 100 nM of FPMXY-14 were treated to Caki-1 and A-498 cells and incubated for 48 h. Further, the coverslips were transferred to new plates, washed with cold PBS, and 2 µl of the combined dye of 100 mg/ml Propidium iodide and 100 mg/ml Hoechst 333258 was added to 20 ml of cell suspension. Coverslips were mounted on glass slides and immediately analyzed with a fluorescence microscope (Nikon, Japan), picturized, and presented.

#### Cell cycle analysis

The assay was carried out with Guava® cell cycle reagent, according to the manufacturer’s instructions. Caki-1 and A-498 cells, at a density of 0.5 × 10^6^ cells per well, were seeded in a 6-well plate and allowed to adhere for 24 h. FPMXY-14 at a concentration of 75 nM and 100 nM were added to Caki-1 and A-498 cells, respectively, followed by another incubation of 48 h. The media was aspirated, and cells were removed by trypsinization. After a couple of washes with sterile PBS, 50 µl cell cycle assay reagent was added, then incubated in the dark for 15 min washed with wash buffer two times and re-suspended in HBSS buffer. Ten thousand events were acquired on a Guava easyCyte™ flow cytometer, and the data were analyzed with ExpressPro Software from Millipore (Burlington, CA, USA). The percentage of cell populations at different cell cycle stages was presented.

#### Annexin V assay

Annexin V detection kit was used for this assay as per the manufacturer’s instructions. 0.5 × 10^6^ of Caki-1 or A-498 cells were grown in 6-well plates and treated with the 75 nM or 100 nM of FPMXY-14, followed by 5% CO_2_ incubation at 37°C for 48 h. After the incubation period, cells were harvested, washed with kit buffer, and incubated with 0.25 µg/ml Annexin V reagent for 15 min in the dark. After a couple of washes, cells were and re-suspended in kit buffer containing 0.5 µg/ml propidium iodide. Ten thousand events were acquired on a Guava easyCyte™ flow cytometer. Data analysis was carried out with InCyte software to differentiate healthy and apoptotic cells s and presented using Graphpad Prism software-version 6.0 (La Jolla, CA, USA).

#### Scratch wound cell-invasion assay

Caki-1 and A-498 cells were seeded in a 6-well plate and grown until confluent. Using a sterile 200 µl pipette tip, approximately a 1-mm channel was made, and the full growth medium was replaced with a serum-free medium containing 50 ng/ml Hepatocyte growth factor (HGF). 75 nM and 100 nM FPMXY-14 were added to Caki-1 and A-498 cells and incubated overnight at 37°C, with 5% CO_2_. Cells were compared to controls under the microscope and photographed.

#### Tumour cell trans-endothelial cell migration assay

Cell migration was analyzed with QCM™ Tumour Cell Trans-Endothelial Migration Assay–Colorimetric kit from Millipore as per the manufacturer’s instructions. Briefly, Caki-1 or A-498 cells with or without different concentrations of FPMXY-14 were allowed to migrate for 12 h through HUVEC cell membrane grown on migration inserts which touched the media in 24 cell culture plate containing 50 ng/ml HGF as chemo-attractant. The migration inserts were removed, gently swabbed, stained, and eluted with kit components. The eluent was transferred to a 96-well stain quantification plate, and the absorbance was measured at 540–570 nm.

#### Western immunoblotting

Western blotting was performed as described elsewhere [19] with slight variations. Kidney cancer cells were plated in 6-well plates at a concentration of 0.2 × 10^6^ cells/ml and incubated overnight at 37°C and 5% CO_2_. After adhering of cells, full growth media was replaced with slow growth media containing 0.04% FBS. After a brief incubation for an hour, desired concentrations of FPMXY-14 were added to the cells along with a DMSO blank and incubated for another 4 h. Cells were lysed with RIPA buffer, and the insoluble materials were removed by centrifugation at 14,000 × g for 10 min at 4°C. 20–40 μg total protein, quantified with Coomassie plus Protein Assay Reagent kit (Pierce; Rockford, IL, USA), were separated by 8%–15% SDS-PAGE. Gel proteins were further transferred onto a nitrocellulose membrane and probed with the respective primary antibodies, followed by the addition of the corresponding horseradish peroxidase (HRP) conjugated secondary antibodies. The membrane was stripped by stripping buffer for 30 min for quantification and normalization with β-actin (1:5000). Protein bands were visualized using ECL reagents (Amersham Bioscience; Piscataway, NJ, USA) and exposed to Kodak X-Omat Blue XB-1 films (Rochester, NY, USA). Bands were quantified using Image J (Ver. 1.46, NIH).

#### Statistical analysis

Each experiment was carried out in triplicate, and results were expressed as mean ±S.D. Statistical analyses were performed using Graphpad Prism 6.0 (La Jolla, CA, USA). GI_50_ and IC_50_ values were calculated using a non-linear regression fit model with variable slope and plotted accordingly. Differences between the two groups were analyzed using the two-tailed Student’s *t*-test, whereas those between three or more variants were analyzed using ANOVA comparisons. Differences with *p* < 0.05 were considered statistically significant.

## Results

### Chemical analysis of FPMXY-14

**Figure 1:**
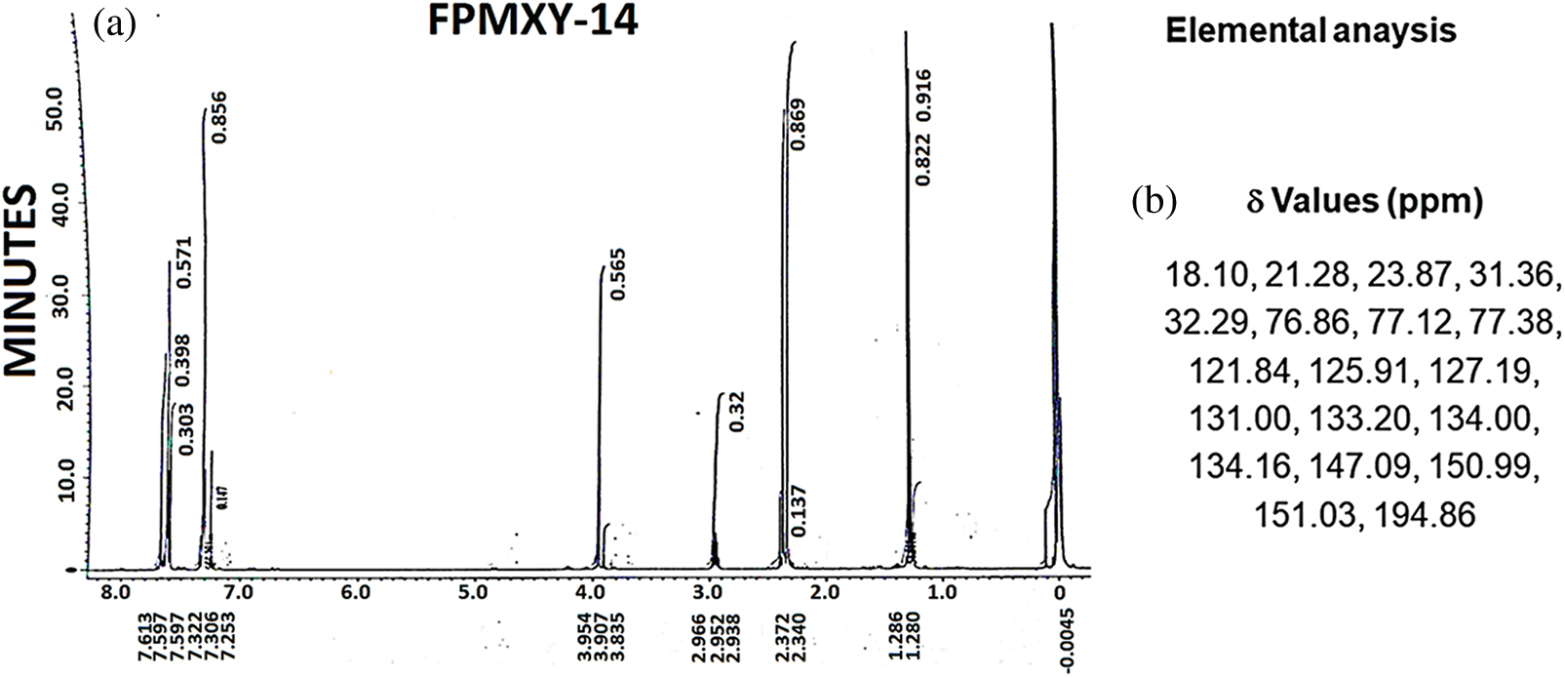
(a) Chemical and structural confirmation of FPMXY-14. (b) δ Values based on NMR results of the synthesized compound.

The IR spectrum (Fig. 1a and Table 1) shows absorption at 1693 cm^–1^ and 1660 cm^−1^, indicating this to be an aromatic α,β-unsaturated ketone. The PMR spectrum shows a multiplet around 7.53–7.70 δ for seven protons (Fig. 1b). These are the six aromatic protons and the one vinylic proton. A singlet at 3.95 δ indicates the benzylic and allylic methylene protons. The single proton for -CH(CH_3_)_2_- appears as a multiplet at 2.95 δ. (Fig. 1b). Two singlets at 2.37 and 2.34 δ for six Ar–CH_3_ protons and a doublet at 1.28 δ for two methyl groups –CH(CH_3_)_2_.

**Table 1:** Elemental analysis depicting physical and chemical properties of FPMXY- based on NMR results

Yield (%)	Melting point (°C)	IR(cm^−1^)	MS (70 ev) M/Z (M+)	Molecular formula	Elemental analysis (%) calcd/found
58	122	1693 1660 1500	290.4	C_21_H_22_O	C H 87.04 7.64

**Figure 2:**
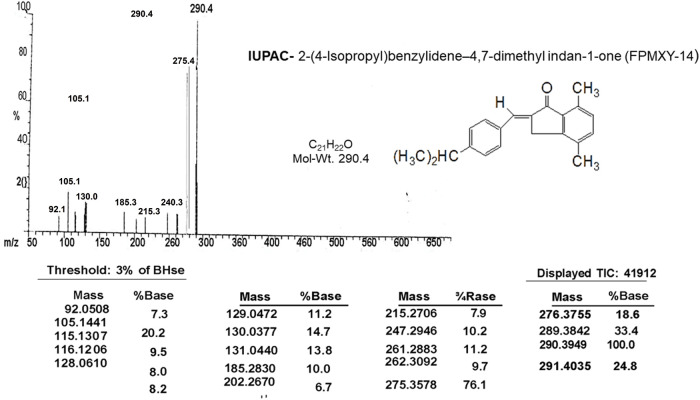
Mass spectrum analysis of the compound confirming the structure and IUPAC name of the synthesized compound.

A signal at 194.86 δ in the ^13^C NMR spectrum (Fig. 2 and Table 2) indicates the presence of α,β-unsaturated carbonyl carbon. The presence of Ar–CH_3_ carbon appears at 18.10 and 21.28 δ. The aromatic carbon atoms appear around 121.8 to 151.03 δ. The m/e value of the compound corresponds to the molecular weight of 290.4 (Table 1) and elemental analysis agrees with the molecular formula of a compound. Calcd: C, 86.96%; H, 7.64%; Found: C, 87.07%; H, 7.64%. (Table 1). Based on the above data, the compound was identified as 2-(4-isopropyl-benzylidene)-4,7-dimethyl-indan-1-one internal reference for the compound FPMXY-14. The compound had 90% purity as determined by 1H NMR.

**Table 2:** Chemical characteristics of FPMXY-14 as analyzed by mass spectroscopy

**M.P.:** 122°C
**IR:** 1689 cm^−1^ – α,β-unsaturated ketone 1650 and 1500 cm^−1^ –C=C and aromatic
**PMR:** 1.28 δ (d, 6H) –CH(CH_3_)_2_ 2.34 δ (s, 3H) – Ar–CH_3_ 2.37 δ (s, 3H) – Ar–CH_3_ 2.95 δ (m, H) – CH 3.95 δ (s, 2H) – CH_2_ 7.53–7.70 δ (m, 7H) –aromatic

### FPMXY-14 selectively inhibited kidney cancer cell proliferations

**Figure 3:**
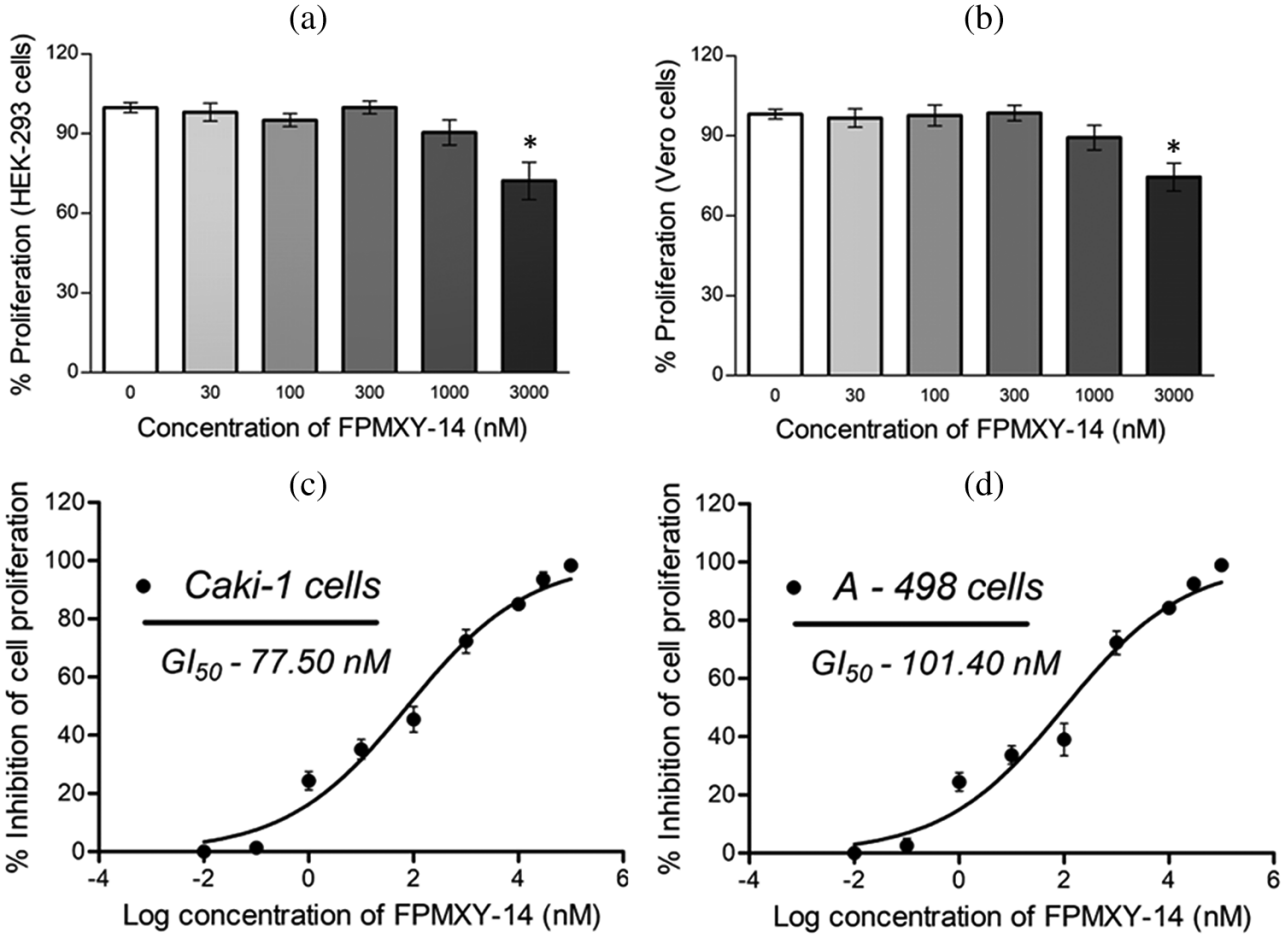
Effect of FPMXY-14 ons normal and cancerous kidney cells. (a) FPMXY-14 showed no toxicity up to 1000 nM in normal (a) HEK-293 and (b) Vero kidney cells. FPMXY-14 dose-dependently inhibited the cancerous (c) Caki-1 and (d) A-498 cells. *p ≤ 0.05 (n = 3) significant compared to control.

**Supplementary Figure 1:**
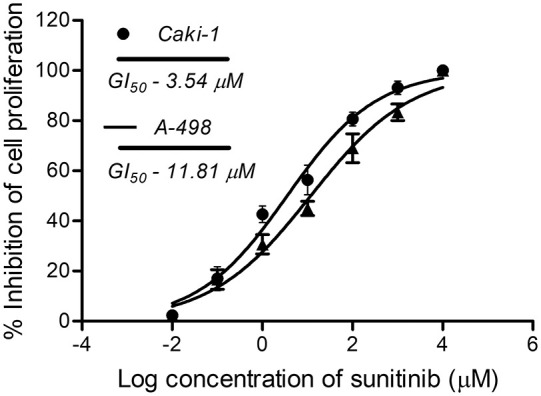
GI_50_ of standard compound sunitinib in Caki-1 and A-498 cells.

Before the test, the compound’s bioefficacy in cancer cells and cell viability was performed for normal kidney cells with FPMXY-14 treatment. The compound showed no adverse effect on the proliferation of human epithelial kidney cell line HEK293 (Fig. 3a) or African green monkey epithelial kidney cell line Vero (Fig. 3b) up to 1000 nM. Further evaluations of FPMXY-14 on cancerous Caki-1 and A-498 cells showed inhibition in the proliferation of these cells in a dose-dependent manner (Figs. 3c and 3d). The compound showed a GI_50_ of 77.5 nM in Caki-1 cells (Fig. 3c) and 101.40 nM in A-498 cells (Fig. 3d). Similarly, the effect of the standard compound sunitinib was tested in both cancer cell lines. Sunitinib inhibited the Caki-1 and A-498 cells at higher concentrations than FPMXY-14 (Suppl. Fig. S1). The GI_50_ concentration of sunitinib in Caki-1 and A-498 cells were 3.54 µM and 11.81 µM, respectively (Suppl. Fig. S1). Unless indicated, the nearest GI_50_ concentration of 75 nM for Caki-1 cells and 100 nM for A-498 cells were used to test the compound’s efficacy in other *in vitro* assays.

### FPMXY-14 bound to the Akt enzyme

**Figure 4:**
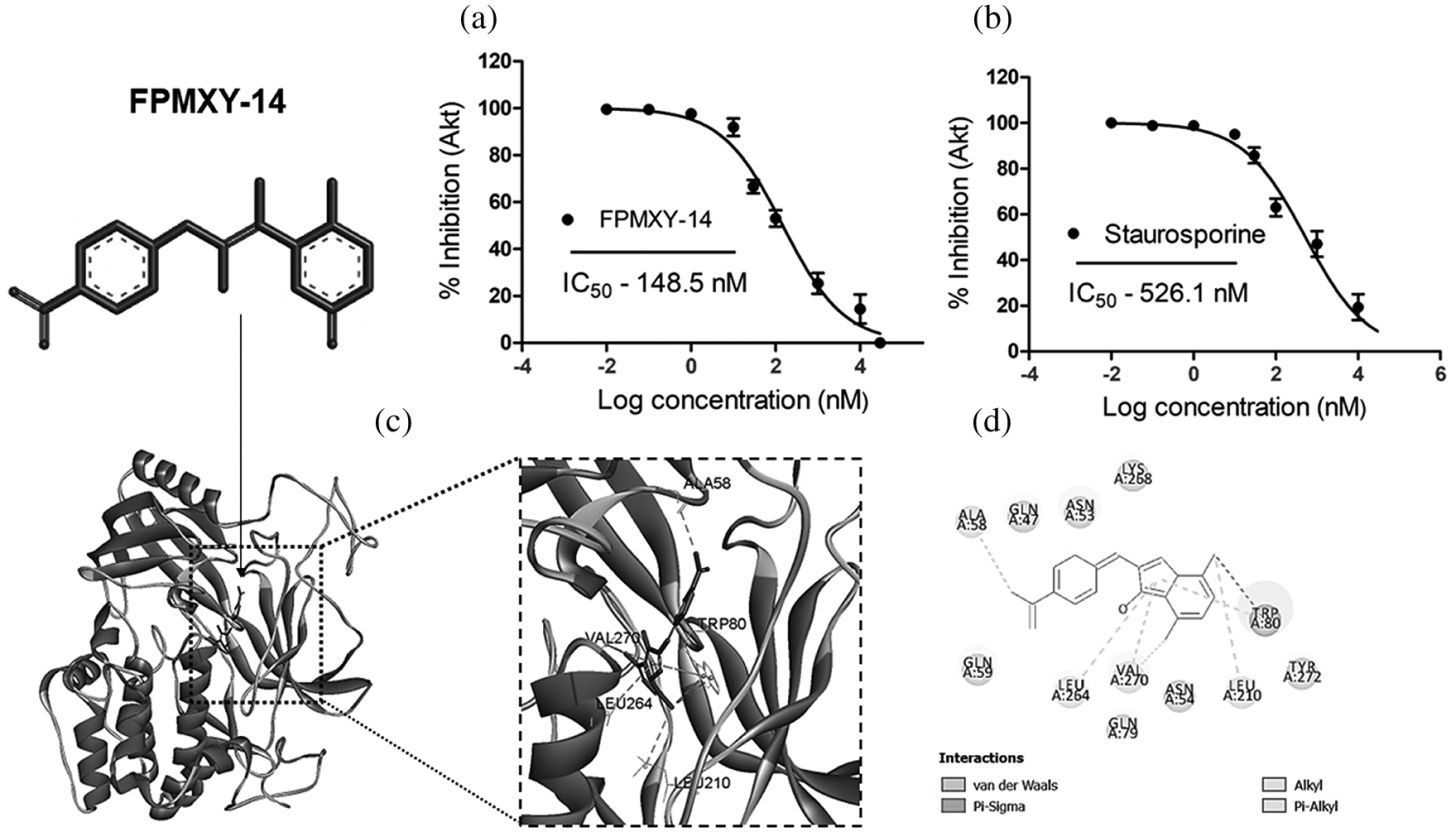
Enzyme inhibitory and Computational docking/MMGBSA analysis of FPMXY-14 with Akt enzyme. (a) The Akt enzyme inhibition assay for FPMXY-14 compared with a known Akt inhibitor (b) Staurosporine. IC_50_ values indicate the inhibitory effect of the compounds (c) FPMXY-14 bound to the allosteric site of Akt and (d) 2D representation of interacting residues of Akt enzyme with FPMXY-14.

Initially, the compound was screened against Akt activity. The compound dose-dependently inhibited the Akt enzyme when tested *in vitro* with an IC_50_ value of 148.5 nM (Fig. 4a). This was much higher compared to the standard compound, which showed an IC_50_ value of 526.1 nM (Fig. 4b). In order to determine the binding target site in the Akt, we performed glide docking followed by prime MMGSBA calculations with FPMXY-14 against the Akt enzyme. Based on our previous studies with arylidene analogues [20], we focused on the allosteric site of the Akt enzyme. Results indicated that FPMXY-14 binds efficiently at the allosteric pocking of the Akt (Fig. 4c). Molecular interactions indicate FPMXY-14 fits efficiently at the allosteric pocket and forms 6 π-alkyl and one π-sigma interactions with Val 270, Trp 80, Ala, 58, Leu 264, and Leu 210 (Fig. 4d).

### Nuclear fragmentation and cell cycle changes by FPMXY-14

**Figure 5:**
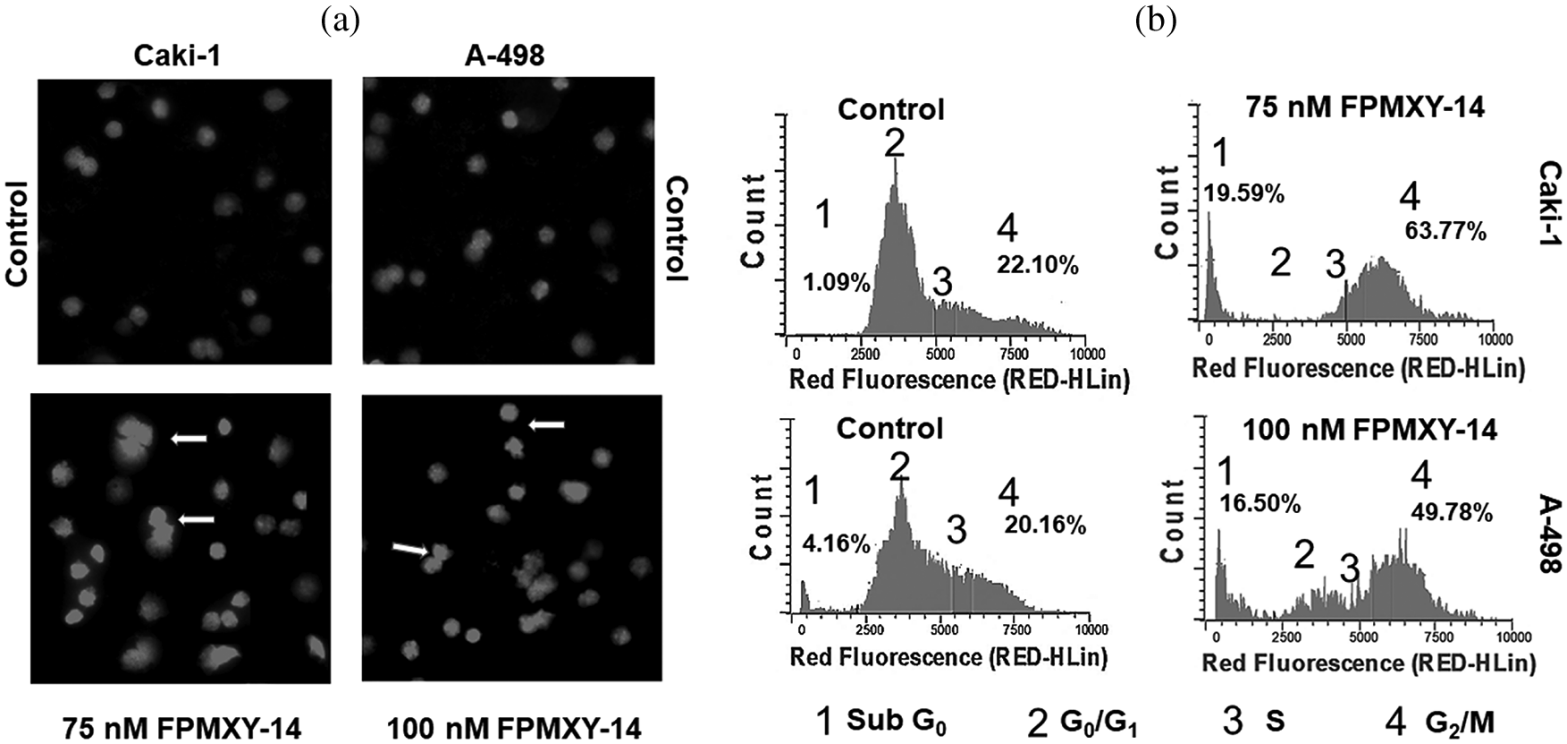
Morphological and physiological evaluation of Caki-1 and A-498 cells treated with FPMXY-14. (a) FPMXY-14 induced nuclear condensation/fragmentation in Caki-1and A-498 kidney cancer cell lines after 48 h treatment as showed by inner arrows. Hoechst 333258 and Propidium iodide are shown in blue and pink pseudo colors, respectively. (b) Flow cytometry analysis of cell cycle changes in Caki-1 and A-498 cells with FPMXY-14 treatments after 48 h. Representative histograms from several repeats of the experiment are shown.

Following compound treatments, nuclear changes were analyzed in kidney cancer cells. At the end of 48 h treatment, FPMXY-14 in both Caki-1 and A-498 cells showed nuclear condensation/ fragmentation similar to apoptotic bodies (Fig. 5a). When analyzed for cell cycle changes, treatment with FPMXY-14 for 48 h resulted in the accumulation of the G_2_/M phase of the cell cycle along with an increment in the Sub G_0_/G_1_ populations (Fig. 5b). 75 nM FPMXY-14 treatment increased the G_2_/M phase to 63.77% from 22.10% of its respective control in Caki-1 cells (Fig. 5b). An increase in Sub G_0_/G_1_ from 1.09% to 19.59% was also observed in these cells (Fig. 5b). Similarly, 100 nM treatment of the compound in A-498 cells resulted in an increase of 49.78% and 16.50% in the G_2_/M phase and Sub G_0_/G_1_, respectively, when compared to control values of 20.16% and 4.16% cells in G_2_/M phase and Sub G_0_/G_1_ phases respectively (Fig. 5b).

### FPMXY-14 induced apoptosis in Cali-1 and A-498 cells

**Figure 6:**
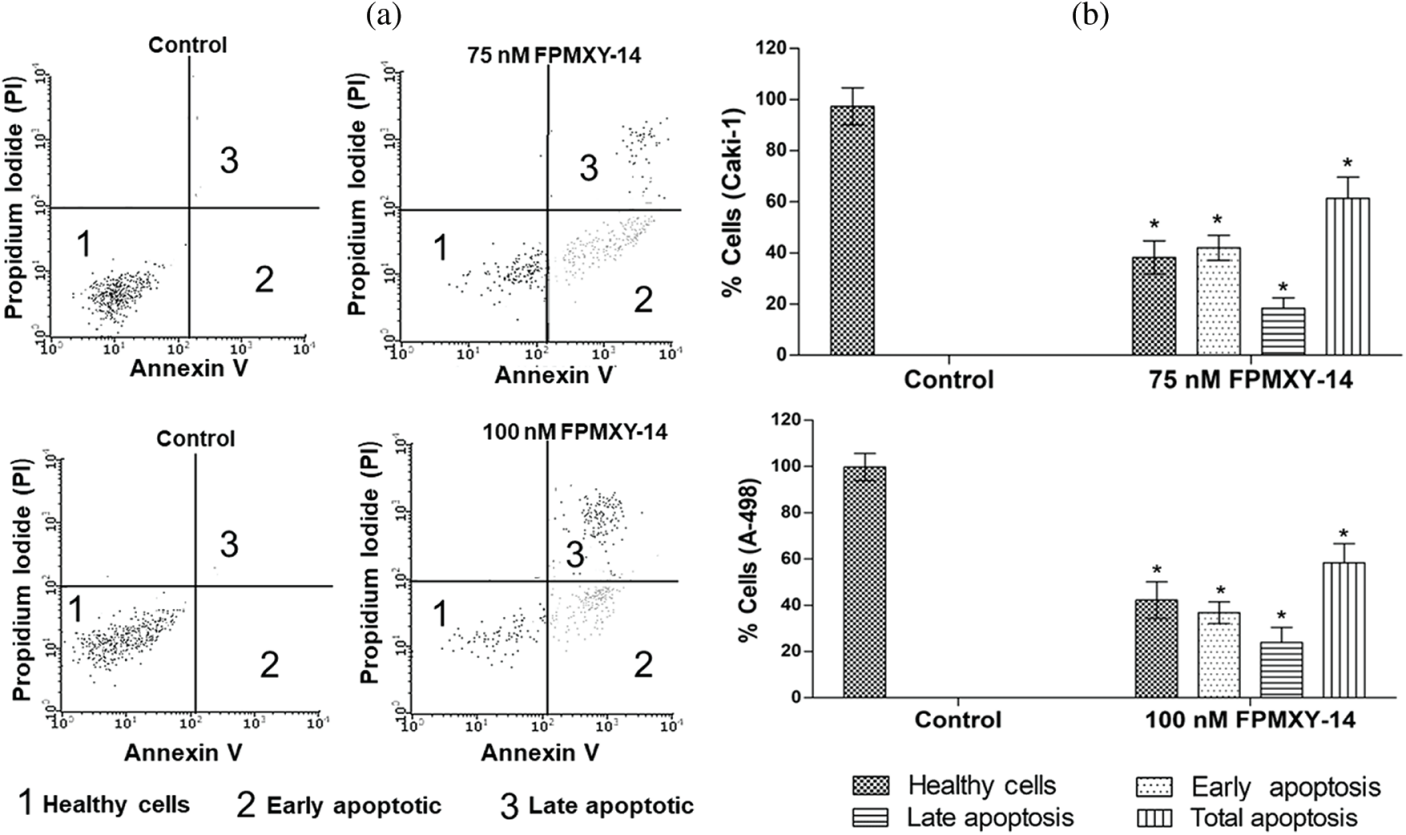
(a) Representative graphs from flow cytometry analysis for apoptosis after Annexin V staining in the kidney cancer cells. (b) FPMXY-14 treatment increased early and late phase apoptotic populations in Caki-1 and A-498 cells. All experiments were performed thrice, and representative results were shown. Results expressed as mean ± SD. Significant *p* ≤ 0.05 (n = 3) compared to * respective controls.

To substantiate our findings regarding nuclear fragmentation and cell cycle changes by FPMXY-14, an Annexin V assay was carried out in both cancer cell types (Fig. 6a). 75 nM compound treatment to Caki-1 cells showed induction of 42.03% early phase and 18.33% late phase apoptotic cells (Fig. 6b). Treatment with 100 nM FPMXY-14 to A-498 cells increased 36.82% early and 21.99% late phase apoptotic cells (Fig. 6b). Total apoptosis was 60.36% and 58.81% in Caki-1 and A-498 cells, respectively (Fig. 6b).

### Anti-metastatic properties of FPMXY-14 in kidney cancer cells

**Figure 7:**
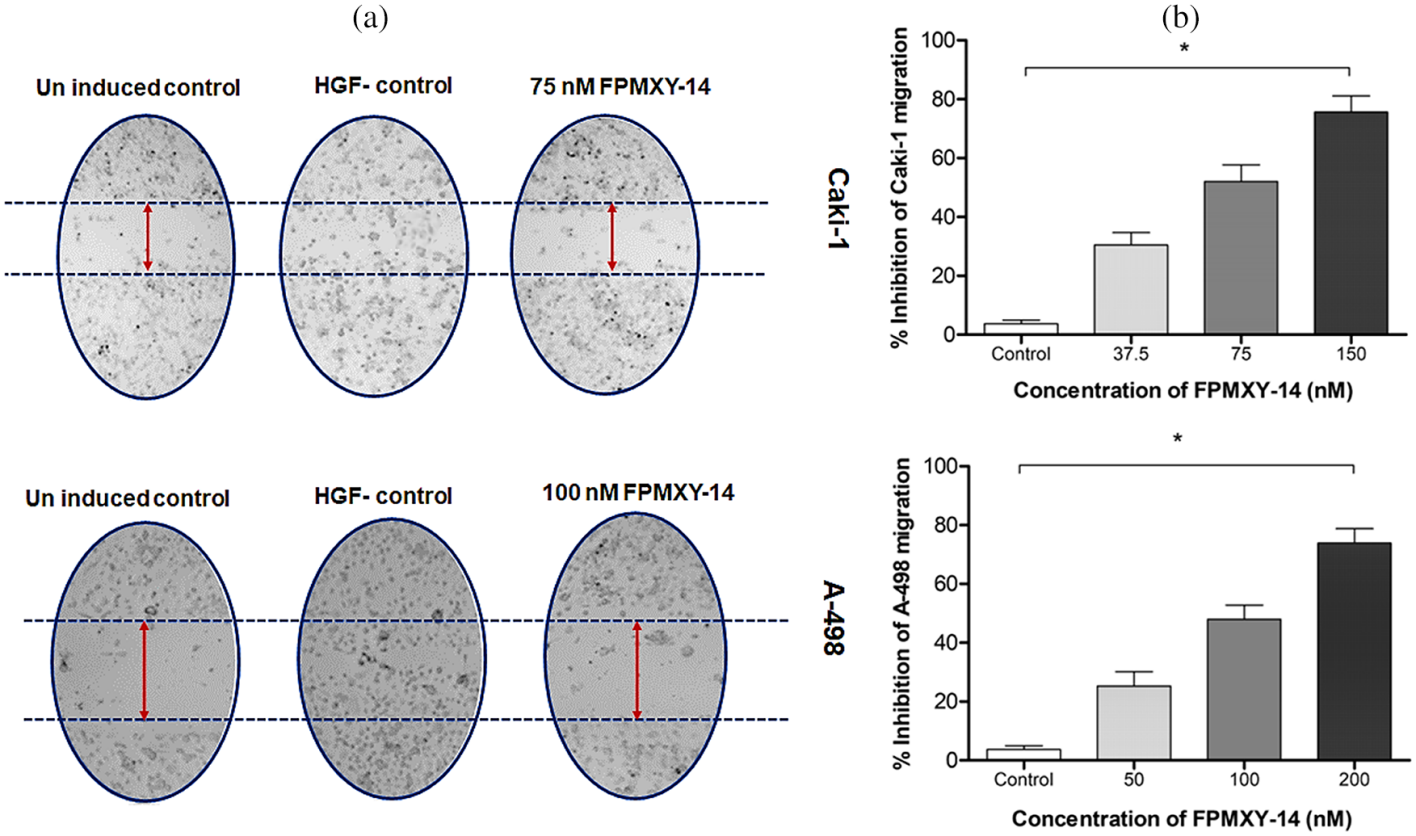
Analysis of anti-metastasis properties of FPMXY-14 in kidney cancer cells. (a) FPMXY-14 was effective against 50 ng/mL HGF-induced scratch wound healing in both Caki-1 and A-498. (b) Anti-migration efficacy of FPMXY-14 on the migration of Caki-1 and A-498 cells across the HUVEC membrane under the influence of 25 ng/mL HGF, which served as chemo-attractant. Results expressed as mean ± SD from three individual experiments in duplicates and *p ≤ 0.05 (n = 3) was considered statistically significant.

To enumerate the effects of FPMXY-14 in the metastasis of kidney cancer cells, HGF-induced scratch wound healing assay and tumor cell trans-endothelial cell migration assays were performed (Fig. 7). Our results demonstrate that treatment with FPMXY-14 inhibited wound healing (Fig. 7a) of both Caki-1 and A-498 cells under the influence of 50 ng/ml HGF. We used the near values of respective GI_25_, GI_50,_ and GI_100_ concentrations of FPMXY-14 in Caki-1 and A-498 cells to check the dose-dependent effect of the compound in the trans-migration assay. As shown in Fig. 7b, FPMXY-14 dose-dependently inhibited the endothelial trans-migration of both the kidney cancer cells across the HUVEC cell membrane under the influence of an HGF.

### FPMXY-14 ameliorated caspase-3 and attenuated Bcl-2/Bax ratio, Akt phosphorylation

**Figure 8:**
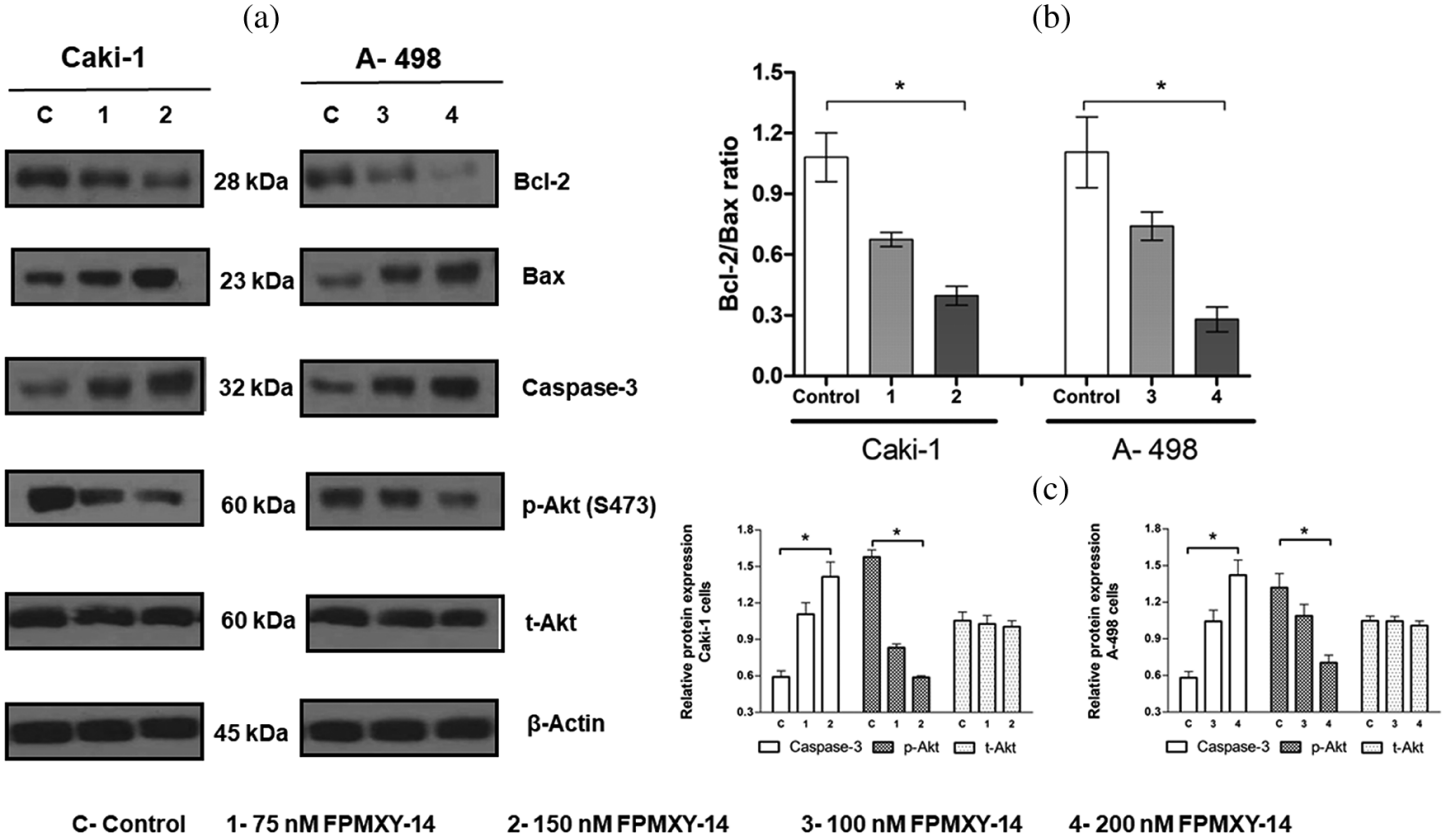
Effect of FPMXY-14 on key signalling proteins of apoptosis. (a) Western blot enumeration for the effect of FPMXY-14 treatments on Bcl-2, Bax, and caspase-3, and p-Akt(s473) expressions in Caki-1 and A-498 cells. Representation blots from three individual experiments are shown. β actin was used as a loading control. (b) FPMXY-14 treatment effectively reduced the Bcl-2/Bax ratio in Caki-1 and A-498 cells. Error bar represents the SD of three independent experiments. *statistically significant at *p* < 0.05. (c) Quantifying the Western blot bands showed a dose-dependent increase of Caspase-3 and a decrease of phosphorylated Akt in both kidney cancer cells when treated with FPMXY-14. Bands were quantified by densitometry using Image J (Ver: 1.46, NIH). After normalizing to beta-actin, results from three individual experiments were expressed as mean± SD and were plotted with GraphPad Prism 6.1. Results were statistically significant at ^#^*p* < 0.05.

To further support the apoptosis induction by FPMXY-14 in the kidney cancer cells, the key signalling proteins were analyzed by Western blotting (Fig. 8). Treatment of FPMXY-14 dose-dependently increased the expression levels of caspase-3 in both Caki-1 and A-498 cells (Fig. 8c). The compound dose-dependently decreased the anti-apoptotic Bcl-2 protein and increased the pro-apoptotic Bax protein levels in both Caki-1 and A-498 cells (Fig. 8a). Subsequently, a decrease in the Bcl-2/Bax ratio for both cell lines was observed as dose-dependent (Fig. 8b). To elucidate the inhibition effect observed in both computation and enzymatic methods, we analyzed the effects of FPMXY-14 treatment on the Akt proteins of both kidney cancer types. Phosphorylation of Akt at serine 473, dose-dependently reduced in both Caki-1 and A-498 cells when treated with FPMXY-14 (Figs. 8a and 8c).

## Discussion

Searching for new chemotherapeutics remains vital in managing RC patients due to resistance anomalies and toxicity hitches acquired in current treatment regimes. The current study focused on screening a novel Arylidene indanone small molecule, 2-(4-Isopropyl) benzylidene–4,7-dimethyl indan-1-one (FPMXY-14), against RC cell types. Small molecules have become more successful chemotherapeutics against cancer cells due to their small size, which enables them to target the extracellular/intracellular proteins and attenuate transduction signals of cell growth and metastasis [21]. The specificity possessed by these molecules has enabled them to be more specific against the targets sparing the non-specific, off-target effects. The anti-proliferative effects observed with FPMXY-14 in Caki-1 and A-498 cells and its non-toxicity towards normal cells at the near-active concentrations were in accordance with the literature for small molecule’s specificity and non-toxicity. Furthermore, the compound was effective at a much lower concentration against the kidney cancer cells compared to the standard compound sunitinib, which proves the higher efficacy of this molecule in controlling kidney cancer cell proliferation.

Targeted therapy remains a prime choice for managing various diseases, including cancer control, due to its established potential in drug discovery [22]. Therapeutic targets for cancer treatment have been looked closely at the molecular level to focus more precisely on their contribution toward tumorigenesis. Akt is a largely focused target on controlling and treating various forms of cancers [23]. Therefore, the current study focused on screening the molecule for its ability to inhibit the Akt enzyme. Arylidene indanone analogs have been successfully screened as targeted therapeutics for various diseases [24]. Our results of Akt inhibition by FPMXY-14 were in line with the reported literature. To further understand the anti-cancer and Akt inhibitory activity of the molecule and confirm the exact binding position of FPMXY-14 with Akt, an *in-silico* computational approach was performed. The molecule is predicted to bind to the allosteric pocket, which opens at the inactive state conformation of the enzyme [25]. Further, MMGBSA calculations identified the binding mode FPMXY-1 to form 6 π-alkyl interactions and one π-sigma interaction with key residues to be responsible for the observed activity against the Akt enzyme [25].

To check the morphology and physiology status of the FPMXY-14 treated RC cells, nuclear staining assay was performed. Propidium iodide and Hoechst 333258 dual-dye stain the fragmented nucleus to indicate condensed/ fragmented DNA within the cells [26]. Such anomalies in the DNA caused by drug treatments frequently indicated the apoptotic mode of cell death [27]. Observations in the current investigation, therefore, suggested apoptotic induction by FPMXY-14 in both Caki-1 and A-498 cells. Cell cycle analysis was carried out to check the physiological status of FPMXY-14 treatment in RC cells. There was an accumulation in the sub-G_0_ and G_2_/M phases of the cell cycle with FPMXY-14 treatments. The characteristic appearance of the sub-G_0_ populations in a cell cycle indicates a loss of DNA content within the cells [13]. Our data, therefore, agreed with the reported literature and was also in line with the nuclear staining data, which indicated a condensed/fragmented nucleus of the Caki-1 and A-498 cells with FPMXY-14 treatments. Reports indicated anti-cancer drugs exhibit cell cycle arrest in the growth phase (G_2_/M phase) while parallelly inducing cell death by apoptosis [28]. Few anti-cancer agents have been shown to damage the cellular DNA resulting in a stagnation of the cell cycle in the G_2_/M phase [29]. Other reports indicated that, while damaged cells progress through the cell cycle arrest in the G_2_/M phase, based on the degree of DNA damage, either they readily enter apoptosis or subsequently pass through aberrant mitosis to finally end up in apoptosis [30]. The observation of the sub-G_0_ population along with G_2_/M phase cell cycle arrest with FPMXY-14 treatments was in favor of the literature mentioned above to suggest early and late apoptosis induction by the molecule in both kidney cancer cell types. These observations were supported by the Annexin V results, which exhibited an increase in the early and late phase apoptosis cells when treated with FPMXY-14 [31]. Further, the current study indicated a dose-dependent inhibition of tumor scratch healing and trans-endothelial migration of Cali-1 and A-598 cells across the HUVEC membrane. These observations stood well with literature indicating the anti-metastatic efficacy of the compound in kidney cancer cells [12].

Classically, apoptosis could be mediated by two pathways. The first one is the mitochondria-mediated intrinsic pathway, which involves a cluster of anti and pro-apoptotic signalling proteins like the Bcl-2/Bax family and the caspases [32]. Another one is the extrinsic pathway that involves the receptor-mediated Akt signalling cascade [32]. Our observations show a clear-dose-dependent increase in the pro-apoptotic Bax levels and a decrease in anti-apoptotic Bcl-2 by FPMXY-14 treatments, ultimately reducing the Bcl-2/Bax ratio. Caspase-3 was also dose-dependently reduced in both Caki-1 and A-498 cells when treated with FPMXY-14. On the other hand, our results also indicated a dose-dependent reduction of Akt phosphorylation (ser473), which was in line with the Akt inhibition results observed in the enzymatic and computational methods. As there is evidence to indicate the occurrence of both pathways to bring out a total anti-cancer effect by chemotherapeutic compound [32,33], it can be postulated that FPMXY-14 would have acted in both ways to attenuate the mitochondrial potential and Akt signalling cascade of Caki-1 and A498 cells, ultimately making them bioenergy deficient and subsequently undergo apoptosis.

## Conclusion

In summary, FPMXY-14 exhibited anti-proliferative and anti-metastatic activities in kidney cancer cells. The molecule exhibited excellent inhibitory activity towards the Akt enzyme by binding to the allosteric pocket in the inactive conformation state. Bio-efficacy of FPMXY-14 against the tested kidney cancer cells was mediated via apoptosis induction. While our results encourage developing this small molecule as a novel chemotherapeutic agent against kidney cancer, further pre-clinical research on animals with a detailed pathway elucidation is recommended.

## Figures and Tables

**Graphical abstract:**
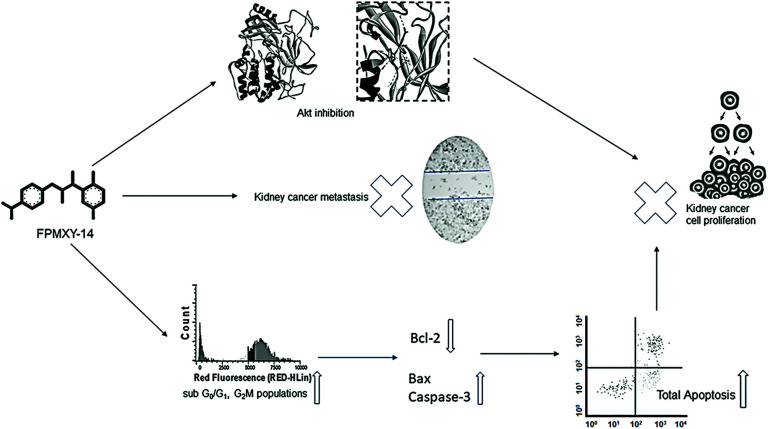
FPMXY-14 exhibited excellent inhibitory activity towards the Akt enzyme by binding to the allosteric pocket in the inactive conformation state. Bio-efficacy of FPMXY-14 against the tested kidney cancer cells was mediated via apoptosis induction.

## Data Availability

All data used in the study were presented here. Any data required is available with the communication author and can be provided upon reasonable request for non commertial purpouses.
